# Metoprolol-induced visual hallucinations: a case series

**DOI:** 10.1186/1752-1947-6-65

**Published:** 2012-02-15

**Authors:** Jonathan A Goldner

**Affiliations:** 1 Department of Medicine, Pocono Medical Center, East Stroudsburg, PA, USA; 2 Commonwealth Medical College, Scranton, PA, USA

## Abstract

**Introduction:**

Metoprolol is a widely used beta-adrenergic blocker that is commonly prescribed for a variety of cardiovascular syndromes and conditions. While central nervous system adverse effects have been well-described with most beta-blockers (especially lipophilic agents such as propranolol), visual hallucinations have been only rarely described with metoprolol.

**Case presentations:**

Case 1 was an 84-year-old Caucasian woman with a history of hypertension and osteoarthritis, who suffered from visual hallucinations which she described as people in her bedroom at night. They would be standing in front of the bed or sitting on chairs watching her when she slept. Numerous medications were stopped before her physician realized the metoprolol was the causative agent. The hallucinations resolved only after discontinuation of this medication.

Case 2 was a 62-year-old Caucasian man with an inferior wall myocardial infarction complicated by cardiac arrest, who was successfully resuscitated and discharged from the hospital on metoprolol. About 18 months after discharge, he related to his physician that he had been seeing dead people at night. He related his belief that since he 'had died and was brought back to life', he was now seeing people from the after-life. Upon discontinuation of the metoprolol the visual disturbances resolved within several days.

Case 3 was a 68 year-old Caucasian woman with a history of severe hypertension and depression, who reported visual hallucinations at night for years while taking metoprolol. These included awakening during the night with people in her bedroom and seeing objects in her room turn into animals. After a new physician switched her from metoprolol to atenolol, the visual hallucinations ceased within four days.

**Conclusion:**

We suspect that metoprolol-induced visual hallucinations may be under-recognized and under-reported. Patients may frequently fail to acknowledge this adverse effect believing that they are just dreaming, or may be embarrassed to report visions that they feel will not be perceived by others to be real. Similarly, healthcare providers can also fail to recognize this visual toxicity or attribute visual hallucinations to concurrent illness or other medications. Clinicians must maintain diligent surveillance when managing patients receiving this drug.

## Introduction

The central nervous system (CNS) adverse effects of beta-adrenergic blockers, including visual hallucinations, have been largely associated with highly lipophilic agents such as propranolol, timolol and pindolol [1,2]. Metoprolol is one of the most commonly prescribed beta-adrenergic blocking agents but visual hallucinations associated with its use have been reported only rarely [3-5]. Here, the cases of three patients with visual hallucinations induced by metoprolol are reported; we review the literature on this topic and hypothesize why this neurologic toxicity may be under-recognized and under-reported.

## Case presentations

### Case 1

An 84-year-old Caucasian woman with a history of hypertension and osteoarthritis suffered from visual hallucinations for several years. She would awake at night to see people standing at the foot of her bed or sitting on a chair in her room watching her sleep. These people would not converse with her but were frightening. She had no history of neurological or psychiatric illness nor were there any significant neuropsychiatric findings on physical examination. Her medications consisted of aspirin, a calcium channel blocker, an angiotensin converting enzyme inhibitor and metoprolol tartrate 50 mg orally twice a day for blood pressure control. She used acetaminophen for control of her arthritis symptoms. She did not have any hallucinations any other time of day or night. A neurological work up included a magnetic resonance imaging of her brain, an electroencephalography and neurocognitive testing, which was unrevealing. A full complement of laboratory tests showed normal thyroid functions, vitamin B12 and folate levels. She had no history of alcohol or drug use. She was unsure how long she had been on all of her medications but knew that she had been taking metoprolol for at least two years. All of her antihypertensive medications were discontinued besides the beta adrenergic blocker before her physician realized the causative agent was metoprolol. Her visual hallucinations stopped completely within several days of ceasing this medication.

### Case 2

A 62-year-old Caucasian man had an inferior wall myocardial infarction that was complicated by cardiac arrest shortly after arrival to the emergency department. He was successfully resuscitated after approximately one to two minutes of ventricular fibrillation with electrical defibrillation. He otherwise had an uneventful recovery and was discharged from the hospital on aspirin, isosorbide, lisinopril and metoprolol tartrate 100 mg orally twice a day. He had no history of neurologic or psychiatric abnormalities or significant findings in this regard on physical examination. He used alcohol rarely but there was no illicit drug use.

About 18 months after discharge, our patient asked to speak to his physician in a confidential manner. He asked that his wife not be told, and subsequently related that he had been seeing dead people at night. He would awake and see faceless figures sitting at the side of his bed; the figures would vanish as he became totally awake. He believed that since he 'had died and was brought back to life' during the acute phase of his myocardial infarction, he was now seeing people from the after-life. He would also see animals at times. The visual hallucinations started immediately after he was discharged from the hospital at which time he had been placed on metoprolol. Upon discontinuation of the metoprolol the visual disturbances resolved within several days. Our patient asked to be restarted on metoprolol after considering the beneficial aspects of the drug on his heart disease. He now understood that the visual hallucinations were related to the medication, and they were no longer frightening to him when they subsequently occurred.

### Case 3

A 68-year-old Caucasian woman who suffered from severe hypertension, hypothyroidism and depression, reported visual hallucinations at night for two years while taking metoprolol succinate 100 mg orally once a day. These included awakening during the night with people in her bedroom and seeing objects in her room that turned into animals. Her other medications included amlodipine, enalapril, escitalopram, levothyroxine and aspirin. When questioned, she believed the hallucinations started about the same time that she was placed on metoprolol for her hypertension. Her physical examination and laboratory evaluation were unrevealing. She had no history of alcohol or illicit drug use. She described the visual disturbances to her previous family physician, who felt this was likely related to her medical problems including her depression. Her neurologic and psychiatric examinations were otherwise unremarkable except for a flat affect. She subsequently visited a new physician who switched her from metoprolol to atenolol, with resolution of her night-time visual hallucinations within four days.

## Discussion

For decades, beta-adrenergic blocking agents have been known to cause adverse CNS effects including psychiatric syndromes, bizarre and vivid dreams, sleep disturbances, delirium, psychosis and visual hallucinations [6,7]. The medical and pharmacologic literature clearly describe the highly lipophilic agents, such as propranolol, as most frequently displaying neurologic toxicities [1,8,9]. In 1978, Fleminger reported an incidence of 14.3% to 17.5% of visual hallucinations and illusions with propranolol among patients attending a hypertension clinic [8].

Metoprolol is a popular cardiovascular drug whose widespread use can be attributed to its generic and affordable short- and long-acting formulations, and its established safety and efficacy in treating a variety of cardiovascular disorders such as hypertension, arrhythmias, angina pectoris, acute coronary syndromes and congestive heart failure. There is a paucity of data on the occurrence of CNS side effects with the administration of metoprolol. Metoprolol, which has an intermediate degree of lipophilicity (a property known to enhance CNS drug penetration, as opposed to hydrophilicity, which limits brain entry), has rarely been reported to cause visual hallucinations despite its common usage [3-5]. In light of our experience, we suspect that the occurrence of visual hallucinations with this drug may occur more frequently than previously reported.

Under-reporting and under-recognition of hallucinations caused by metoprolol might be due to several factors. First, patients may not complain of visual disturbances because they fail to connect the symptoms with the drug, as in our second case. Some patients may attribute beta-blocker-associated hallucinations to dreaming or nightmares [8]. As Fleminger reported with propranolol, all three of our patients had their visual hallucinations while awakening from sleep in the hypnopompic state [8]. Furthermore, patients may be too embarrassed to discuss illusions that they fear may be confused by others as being a result of mental illness or excessive drug or alcohol use. Finally, physicians may also fail to recognize this adverse drug effect and may consider the visual disturbance to be related to existing medical or psychiatric conditions, as in our first and third case.

Sirois reported that hallucinations associated with metoprolol may remain an isolated symptom, or they may evolve into delirium in older patients with cognitive deficits [5]. For both of the patients he described, visual hallucinations began within 24 hours of the initiation of metoprolol and the side effect was discovered shortly thereafter during their hospitalization. This is unlike our patients, who did not recognize the adverse reaction themselves or have it recognized by their physician for a long period of time. The delay in diagnosing the cause of the hallucinations in our patients was either related to the patient's reluctance to inform anyone of the visual disturbances, the patient's family associating the hallucinations with other medical or psychiatric disorders they thought the patient had or the physician's inability to recognize the medication as the etiology for the disorder. Our patients had no visual disturbances during other times nor a psychiatric or medical reason that would otherwise account for the hallucinations. The hallucinations in the cases described were realistic enough to cause considerable anxiety and fear for these patients. Most hallucinations caused by beta-adrenergic agents typically stop within a few days of drug discontinuation, as it did in our patients [8,9]. Depending on their diagnosis, patients with hallucinations related to metoprolol may tolerate a switch to a more hydrophilic beta-blocker such as atenolol or bisoprolol [4].

Interesting, the newer third generation of beta-blockers such as nebivolol and carvedilol, which are frequently used to treat those patients with congestive heart failure and hypertension, thus far have not been associated with visual hallucinations despite their moderate to high lipophilic nature. Thus it is assumed that there may be other factors that may influence the degree of CNS side effects of beta blockers other than lipophilicity. Other parameters, such as the specific structural details of the beta-blocker molecules, drug-induced increases in plasma catecholamine levels and a decrease in melatonin levels have all been proposed to affect the beta-blocker penetration of the blood-brain barrier or the occurrence of CNS side effects [10-12]. These third generation beta-blockers may be a good alternative to metoprolol if visual hallucinations occur.

## Conclusions

Metoprolol, a widely used beta-blocker, has been associated with visual hallucinations and CNS disturbances. Multiple reasons may lead to under-recognition and under-reporting of this adverse drug effect, by both patients and physicians alike. The true incidence of visual hallucinations related to metoprolol is unknown. Clinicians are urged to maintain diligent surveillance when managing patients receiving this drug.

## Consent

Written informed consents were obtained from the patient or next of kin for publication of this case series. Copies of the written consents are available for review by the Editor-in-Chief of this journal.

## Competing interests

The author declares that they have no competing interests.
